# Evaluation of Quality Indicators of Breast Cancer Management at a Tertiary Cancer Center in Nepal

**DOI:** 10.1200/GO.21.00303

**Published:** 2022-03-17

**Authors:** Shweta Baral, Sudhir Raj Silwal, Utsav Man Shrestha, Deep Lamichhane

**Affiliations:** ^1^ Clinical Oncologist, Bhaktapur Cancer Hospital, Bhaktapur, Nepal; ^2^ Surgical Oncologist, Bhaktapur Cancer Hospital, Bhaktapur, Nepal

## Abstract

Breast cancer is the second commonest cancer among female in Nepal. This is our first attempt to audit breast cancer management in our institute and compare with standard quality indicators (QIs) available.

**METHODS:**

The retrospective study included 104 female patients with breast cancer who had taken treatment at Bhaktapur Cancer Hospital in 1 year. Participants were selected on the basis of convenience sampling. Of 33 QIs in breast cancer management according to European Society of Breast Cancer Specialists guidelines, 19 QIs were chosen relevant to our setup. These QIs were calculated for all patients and compared with the European Society of Breast Cancer Specialists standard target. Frequencies and percentages were calculated and presented in tables. Binomial 95% of the rates for QI adherence were also calculated for each QI.

**RESULTS:**

One hundred four patients had a median age of 47.5 years (range 24-70 years). Applicable QIs were in the range of 5-15 with a mean of 9.66 per patient. Of 19 evaluable QIs, very high adherence rates were observed in six QIs, high adherence in three Qis, and low adherences in 10 QIs. High adherence rates were for QI 5 and QI 10a, which were 88.46% and 94.73%, respectively. The low compliance was for QI 1, QI 4a, QI 8, QI 9d, QI 10b, QI 11a, QI 11b, QI 13b, QI 13e, and 14b, which were 53.84%, 78.21%, 0%, 83.16%, 76.92%, 36.0%, 33.33%, 4.76%, 30.55%, and 10.81%, respectively.

**CONCLUSION:**

There are several QIs that have low levels of adherence in our setting and suggest that there is significant room for improvement. We will be continuing auditing these QIs regularly to improve our quality of care.

## INTRODUCTION

Breast cancer is the second commonest cancer among female in Nepal. The GLOBOCAN has estimated that there were 20,508 new cancer cases and 13,629 cancer deaths in Nepal in 2020, of which breast cancer is the fourth commonest cause of cancer-related death (both sexes, 7.7%).^1^ Our hospital had registered 345 patients with new breast cancer in 2019 and 260 patients with new breast cancer in 2020, accounting for 10.09% and 10.3% of all new cancer cases, respectively.

**CONTEXT**

**Key Objective**
Quality indicators are standard methods of assessment of management at any institution, of which our hospital has adopted 19 quality indicators in the management of breast cancer relevant to our setup. Continuing measurement and monitoring these quality indicators might improve our quality of care in breast cancer in the future.
**Knowledge Generated**
Inadequacy of documentation of staging workup, very low compliance to neoadjuvant chemotherapy and targeted therapy, low rate of breast conserving surgery, and absence of multidisciplinary team discussion before management are our key findings of our study.
**Relevance**
We have identified significant rooms for improvement. The causes of low compliance can be addressed on the basis of clinical and system- and patient-level factors. Proper recording of staging investigation and establishment of multidisciplinary team will be the initial steps for improvement of care. Identification and management of patient-related factors with involvement of stakeholders for policy-making work may further supplement the need of project.

Treatment for breast cancer involves multidisciplinary care across modalities like surgery, pathology, radiotherapy, and systemic therapy. As the care process is complex, various clinical practice guidelines are available to ensure that optimal care is provided.^2-5^

The European Society of Breast Cancer Specialists (EUSOMA) proposed a list of 33 benchmark quality indicators (QIs) in 2010 to allow standardized auditing and quality assurance of care provided at local and national levels.^6^ The list of QIs was updated in 2017 to encompass new developments in diagnosis and treatments. Variability in the compliance with these QIs has been reported in several national and regional audits.^7-9^

Our hospital is a public sector hospital where cancer treatment is provided at highly subsidized costs. Despite this, there is a significant out-of-pocket expenditure for a substantial number of patients. In addition, patients availing treatment have widely disparate education and social and economic profiles. Hence, it is expected that there will be variability in adherence to clinical practice guidelines in our setting. The primary objective of the study is to audit the breast cancer treatment data of our institute and determine the adherence to selected EUSOMA QI in our institute. We aim to use these data to formulate hospital-level guidelines for enhancing uniformity of cancer care, audit the quality of care, and incorporate them in our newly formulated hospital-based breast cancer treatment guidelines.

## METHODS

### Study Design

Cross-sectional study—medical audit.

### Setting

We reviewed outpatient department (OPD) clinical record files of patients who were registered at our hospital between April 14, 2019, and April 13, 2020.

### Participants

Formal random sampling was not performed. A convenience sample of all patients with a diagnosis of breast cancer who were registered in the hospital between the aforementioned dates were used. Patients were selected on the basis of availability of their record files. Patients who had not received treatment at our center (and had come for a few visits for opinion only) were excluded. Also, patients with multiple primary tumors and metastatic disease were excluded from the study. Patients whose chemotherapy or targeted therapy were ongoing were excluded. Ongoing endocrine therapy was allowed.

### Variables and Outcome Measures

QIs chosen for this study were taken from the EUSOMA guidelines published in 2010.^6^ A series of meetings were organized between the radiation oncology, medical oncology, and surgical oncology departments in our hospital in which these QIs were discussed individually. We included 19 QIs on the basis of their relevance to our setup. Four (4) QIs were excluded as they pertained to investigations and procedures not available at our center. In addition, as the focus of this audit was to evaluate the quality of treatment provided, QIs related to follow-up and rehabilitation such as appropriate follow-up, availability of nurse counseling, and data manager were excluded.

Sentinel lymph node biopsy is not possible in our hospital because of unavailability of frozen section equipment. QI included pertained to staging workup (QIs 1, 14a, and 14b), preoperative diagnosis (QI 3), completeness of prognostic/predictive marker characterization (QIs 4a and 4c), waiting time for primary treatment (QI 5), multidisciplinary discussion (QI 8), appropriate surgical approach (QIs 9a and 9d), postoperative radiotherapy (QIs 10a and 10b), avoidance of overtreatment (QIs 11a and 11b), appropriate endocrine therapy (QIs 12a and 12b), appropriate chemotherapy, and other medical therapy (QIs 13a, 13b, and 13e).

For staging workup, we had reviewed whether all patients had undergone pretreatment staging investigations including chest x ray, ultrasonography of abdomen, and bone scan wherever indicated. Clinical stage was extracted from the OPD record file. All cases were reviewed for completeness of preoperative pathologic diagnosis and completeness of prognostic/predictive markers. Data of histopathologic type; grading; estrogen receptor (ER), progesterone receptor (PR), and human epidermal growth factor receptor 2 (HER2) status; pathologic stage; size of the invasive component; and margin status were abstracted. Additional details retrieved included waiting time for primary treatment (either surgery or preoperative chemotherapy) measured from the date of registration at hospital, type of surgery, radiotherapy, and systemic therapy.

QI adherence was calculated on the basis of the provided definitions for each QI for each patient. If data were missing, then QI was considered to be nonadherent. All the data were compiled and recorded in a spreadsheet. At first, total numbers of QIs applicable to each patient were calculated (eg, for patients receiving hormonal therapy in hormone-sensitive tumors, with the same QI not applicable to ER−,PR– patients). Then, QIs adhered were calculated for all. Patients were categorized into different groups on the basis of age, grade, stage, etc, and for them, mean and range were calculated. The QI adherence rate for each QI was calculated by dividing the number of patients for whom the QI adherence was documented by the total number of evaluable patients. Adherence to QI was considered as very high when compliance was above the standard target, high when compliance was between the minimum standard and the target, and low when compliance was below the minimum standard. Reasons for lack of adherence to QI were also evaluated in terms of the patient's factor, institutional factors, physician's preference, and unknown causes

In addition, for each patient, we calculated the total number of QIs applicable for that patient and the total number of these QIs adhered to.

### Statistical Analysis

Summary statistics for numerical data included the median with range. For categorical data, frequencies and percentages were calculated. Binomial 95% of the rates for QI adherence were also calculated for each QI. Descriptive summary of the reasons for lack of adherence is provided. In addition, summary statistics (mean and range and standard deviation) are provided for the number and proportion of applicable QIs adhered per patient. In addition, these were calculated for important pretreatment patient characteristics. However, given the retrospective nature of the audit and the small sample size, formal statistical tests for associations were not conducted.

### Ethics Statement

Research approval was obtained from the Nepal Health Research Council institutional review board and hospital administration before conducting the study.

## RESULTS

Three hundred thirty-two (332) patients with new breast cancer were registered in 2019/2020 (2074 BS—Nepal year) during the study period. Of these, 56 patients were excluded as they did not take further therapy, 40 patients had metastatic disease, and for 13 patients, OPD record files were lost. After further exclusion of 119 patients whose treatment was ongoing at the time of this audit (systemic therapy or targeted therapy), 104 patients were evaluable. Characteristics of the 104 patients are presented in Table 1. All patients were female. Compliance with individual QI is shown in Table 2.

**TABLE 1:**
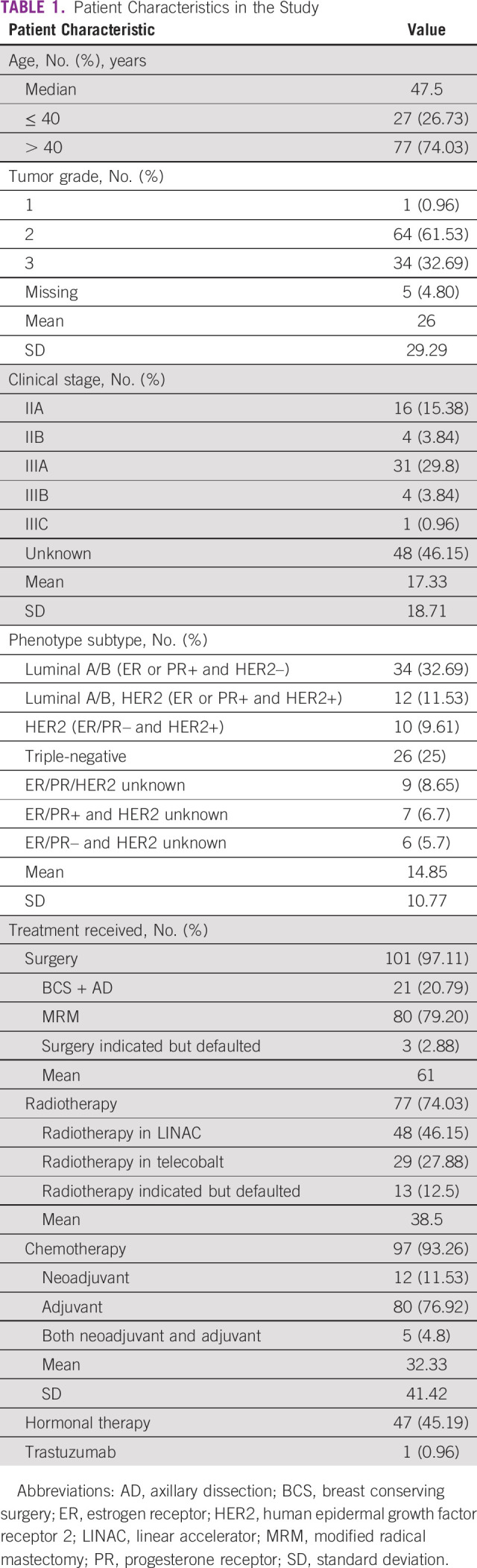
Patient Characteristics in the Study

**TABLE 2:**
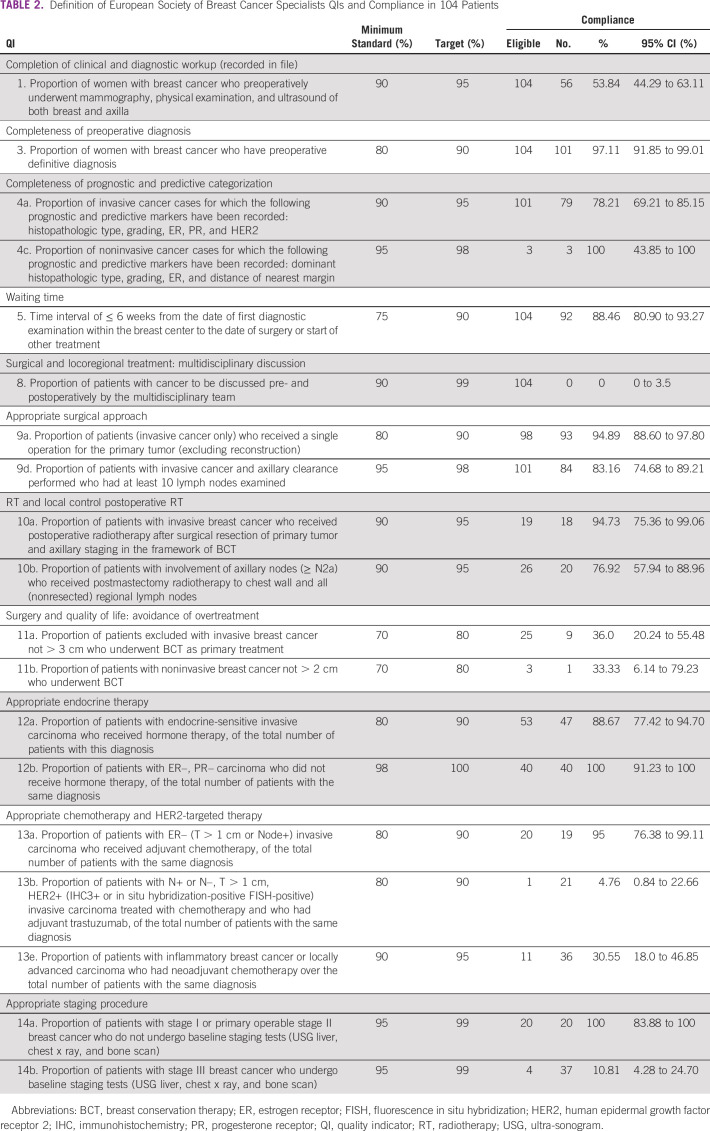
Definition of European Society of Breast Cancer Specialists QIs and Compliance in 104 Patients

### Pretreatment Evaluation and Workup

Of the four QIs related to pretreatment evaluation, low adherence was seen for two QIs. The reason for lack of adherence to QI 1 was unknown/missing records from clinical files for clinical staging workup. In most of the cases, clinical examinations at first assessment were not recorded in the OPD record file and copies of mammogram and ultra-sonogram axilla were missing from the file. For QI 14b, prestaging workup was not completed in 33 patients (89.19% patients with stage III or more). This is because of the unavailability of investigations (bone scan) at our hospital and patient's cost issues.

### Surgery

Low adherences were in five QIs of six QIs for surgery. The waiting time to start treatment was in the range of 2-62 days. Twelve (11.88%) patients in our study had their primary treatment started after 6 weeks of diagnosis, most common cause being patient factor (financial and logistic issues—arrangement of government funds and temporary stay nearby the hospital). An established breast multidisciplinary team (MDT) multidisciplinary discussion was missing in all cases (QI 8).

The reason for inadequate axillary dissection in 18 of the 101 patients remains unknown and undocumented. The compliance for breast conservation therapy in our setup is only 36% for tumors < 3 cm in invasive breast cancer cases and 33.33% in tumors < 2 cm in noninvasive cancers. The values are far less than the minimum target to be achieved. This was because of preference of treating surgeons and choice of patients related to their educational and socioeconomic status. Patients usually choose modified radical mastectomy (MRM) as their perception of less chance of recurrence after MRM and chances of avoidance of postoperative radiotherapy in MRM cases, which ultimately reduces their total cost of treatment.

### Completeness of Prognostic/Predictive Markers

Low adherence was found in one QI of two QIs in the completeness of prognostic/predictive markers. For 80 patients of 101 invasive breast cancer cases, all prognostic and predictive markers were recorded in the file. Twenty-two patients had data of histopathologic type and grade of disease, but data were missing for ER/PR and HER2 status. The reason behind this is the unavailability of immunohistochemistry for the examination of ER/PR and HER2 status at our center and usually sent outside after a histopathologic report.

### Radiotherapy

Low adherence was recorded in postoperative radiotherapy in tumors with ≥ N2a disease after MRM. Seven patients defaulted treatment for whom post-operative radiotherapy after MRM (≥ N2a) was indicated. Four patients defaulted to radiotherapy after chemotherapy, two patients defaulted to adjuvant treatment after surgery, and one patient defaulted after neoadjuvant chemotherapy and surgery. The primary reason for default in radiotherapy is the long waiting time for radiotherapy (3 weeks to 2 months) in our hospital and the patient's logistic and financial factors.

### Systemic Therapy

Low compliance was in three of four QIs in systemic treatment. Of 21 HER2-positive cases in our study, only one patient (4.76%) had received adjuvant trastuzumab, whereas none could afford neoadjuvant trastuzumab. The finding is the result of various factors, with most important being the remarkably high cost of trastuzumab in our country. Compliance for neoadjuvant chemotherapy for the indicated patients is only 30.55% in our study. This may be because of a patient's anxiety to undergo surgery as soon as possible after diagnosis, sometimes physician's preference, and lack of MDT discussion before management in our setup.

Six (11.32%) hormone-sensitive patients defaulted endocrine therapy after surgery. One of six patients of clinical stage IIIC defaulted after neoadjuvant chemotherapy and surgery, one patient defaulted after surgery for further adjuvant treatment, and other four patients defaulted after chemotherapy. The result of low compliance is only patient factors related to logistics.

### Patient-Specific QI Adherence Rate

Applicable QIs were calculated for all patients on the basis of QI criteria (either ER/PR+ or –, HER2+ or –, tumor size ≤ or > 3 cm for invasive tumors and ≤ or > 2 cm for noninvasive tumors, staging of tumor, and other related factors).

The number of QIs adhered per patient ranged between 2 and 10 with a mean of 6.88. Applicable QIs were in the range of 5-15 with a mean of 9.66 per patient. The percentage of applicable QI indicators adhered to range between 33.33% and 90.90% with a mean of 69.80%.

Table 3 shows the distribution of the QI adherence rates with pretreatment patient characteristics.

**TABLE 3:**
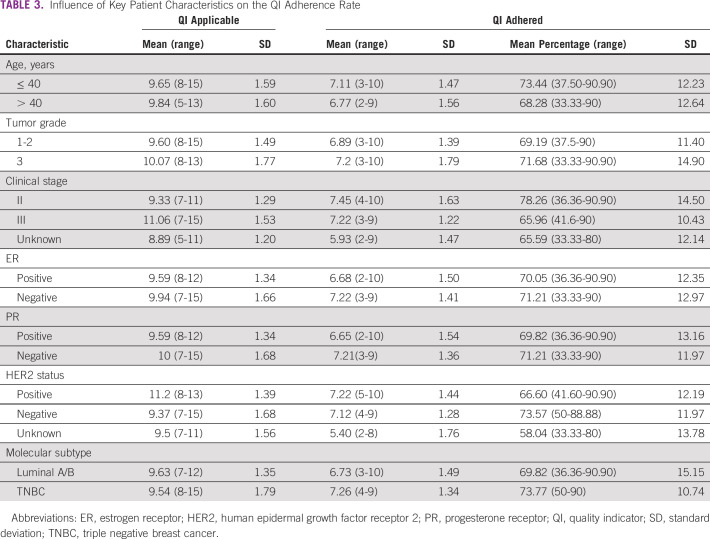
Influence of Key Patient Characteristics on the QI Adherence Rate

### Factors Influencing Low Compliance to QIs

Patient-related factors like financial and educational status and other logistic factors including arrangement of temporary stay nearby the hospital and lack of family support have influenced choices of patients regarding the line of management. Relatively high costs for chemotherapy and trastuzumab, costs for management of side effects, availability of family members/caregivers to take care of patients during treatment, and costs for stay of patients and caregivers around hospital during the length of treatment are the primary causes of low compliance of patients for the completion of treatment. Poor educational status of women, lack of priority to women's health, and awareness of cancer management further credit to low adherence to treatment.

Few investigations like immunohistochemistry for hormonal status markers and bone scan are not available at our hospital, which not only increases the cost and timeline of management but also increases the chance of missing those investigations during management. MDT for breast cancer treatment is not established here. We do’ not have our hospital-based guideline or checklists of the essential examination and investigations during workup. Investigation reports are recorded in OPD record files. Retrospective review of these files revealed missing records of investigations required for proper clinical staging of tumors, which is one of the major causes of low compliance of staging workup in our study. In addition, lack of government insurance policies to cover cancer treatment in our setup and deficit of awareness programs for proper guidance of cancer management prioritize patient's choice of treatment.

## DISCUSSION

The primary purpose of the study was to obtain baseline data for a prospective quality improvement study where we plan to have a uniform institutional protocol for all patients with breast cancer. The QIs that are reported have been included in our protocol and will be regularly audited as a part of a quality improvement study. As the current audit suggests, there are several QIs that have low levels of adherence in our setting.

Bhaktapur Cancer Hospital is a 125-bedded government-funded, comprehensive cancer hospital established in 1992 and provides oncologic services to approximately 20,000 patients with cancer every year. It is also the only government cancer center in the city with radiotherapy facilities. As a consequence, a large number of patients get registered for radiotherapy alone. Most of the patients registered are of low and middle socioeconomic status. A large proportion of patients hail from outside of the city as there are only two centers outside of Kathmandu that provide all oncologic services.

Most of the cancer treatment is also self-funded as there are no established government or private health care insurance policies. The government does provide financial support to the tune of 100,000 Nepalese rupee ($800-$900 US dollars) for all patients newly diagnosed with cancer. However, the total cost of breast cancer treatment is about $2,500-$3,000 at our hospital excluding charges for investigations and the cost of trastuzumab when needed. With a per-capita annual gross domestic product of $1,155 (2020, World Bank data),^10^ there is a significant out-of-pocket expenditure.^11^ The cheapest trastuzumab (biosimilar) available in the country costs around $5,000-6,000 for the 1-year course.

There are several studies which showed that clinical practice guidelines improve quality of treatment. In these studies, centers had compared QIs before and after institution adherence to the practice guidelines.^12^ Few European hospital–based studies had shown that QIs are useful tools to evaluate care of organizations.^13,14^

Similarly, a set of QIs were identified in a Spanish study that served as a basis of strategy for benchmarking oncology services across Spanish hospitals to improve quality of care.^15^

In a Norwegian study, clinical breast cancer registry data from 2012 to 2016 were used to estimate QIs. Increased compliance to recommended treatment has been observed during the registry years. The registration of treatment administered at all hospitals made it possible to make changes and follow treatment after implementation of new guidelines.^16^

The Breast Health Global Initiative group had developed resource-stratified guidelines for breast cancer management, which divided the health care delivery system into four tiered systems on the basis of available resources.^17^ The guideline was formulated to address the resource constraints in low-middle–income countries (LMICs) and to improve health care delivery systems by setting the basic level of standard in practice with respect to the tiered system. As per investigations, our hospital lies in the limited setting group because of unavailability of specimen radiography and bone scan at our center. Sentinel lymph node mapping and biopsy are not performed, which moves surgical treatment to the basic level of management. On the basis of other available resources here, radiotherapy and systemic treatment are in the maximal group. Because of these disparities, it has become difficult to compare care on the basis of the stratified guideline in our institute.

Comparing preoperative investigations and diagnosis with a limited resource level of the stratified guideline, the standard of QIs is matched up to the target level, but in the same setting, there is low compliance for QIs in systemic therapy and radiotherapy group when compared with the maximal resource level. The QI of postoperative radiotherapy in the MRM group is only 76.92%, and that of neoadjuvant chemotherapy is 30.55% in our study.

Breast Health Global Initiative 2018 highlighted the clear need of systematic and strategic approaches to translate resource-stratified guidelines into clinical use.^18^ Stakeholder's identification, situation analysis, cancer control planning, and phased implementation of strategies are the important steps in implementing resource-stratified guidelines in LMICs.

Identification of a patient's pathway to treatment, addressing the causes of delay in treatment, and lack of participation in management play a very important role in managing patient-related factors.

Our study also suggests that there is significant room for improvement. On the basis of the result of this audit, we have further discussed several mitigation strategies to improve adherence rates to QI. Some of these mitigation strategies are implementable at an institutional level, whereas others would require engagement with stakeholders in the population and government.

As one of the key findings of the study, only 53% had their clinical stage recorded before starting treatment. This might affect the overall outcome of the treatment including disease-free survival and overall survival. We had excluded patients with metastatic disease. However, some patients with unknown staging status might have had metastatic disease. In the absence of documentation, we are unable to ascertain this further. The obvious implication of treatment with metastatic disease is that patients might not have had the stage-appropriate systemic therapy or received local therapy, which was not warranted. More rigorous attention to staging and documentation is therefore essential.

One of the key mitigation strategies that we plan to implement is to initiate a process for MDT discussion for breast cancer cases. This is likely to affect five QIs directly and four QIs indirectly. A study by Taylor et al^19^ in 2013 showed that MDT-based care is superior to improve quality of care in patients with breast cancer. MDT practice has strong clinical evidence to support its utilization, with an increase in diagnostic accuracy, treatment planning, and patient health outcomes.^20^ Different studies have shown high compliance with MDT before starting management in breast cancer.^21,22^ We expect that systematic implementation of a multidisciplinary tumor board meeting would enhance uptake of neoadjuvant chemotherapy and breast conservation in our practice.

Another key institutional mitigation strategy is implementation of hypofractionation for most patients. Hypofractionated radiotherapy has been demonstrated to be noninferior to conventional fractionation.^23,24^ In addition, real-world data from our neighboring country demonstrate the real-world effectiveness of this strategy.^25^ Hypofractionation is expected to shorten overall treatment time, make treatment more affordable, reduce waiting time, and improve access to radiotherapy for all patients.

In the absence of an institutional electronic medical record system, retrieval and archival of test results performed outside remains a challenge. The low adherence to the two QIs in the pretreatment workup and evaluation is a direct result of inadequate documentation and lack of facility for record storage. Introduction of an electronic medical record system would require substantial investment at an institutional level. However, work of Raut et al^26^ suggests that adoption of an open-source electronic medical record may be feasible in our setting. In the meantime, we plan to design a standardized case record form to improve adherence to these QIs.

ER and PR status was recorded in noninvasive tumors up to the standard level, but for the invasive cancer, the compliance was only 78.21%. Studies have shown that prognostic and predictive markers play a very important role not only in appropriate endocrine therapy management but also in determining prognosis and planning for chemotherapy (neoadjuvant/adjuvant, agents) and timing and choice of surgical management in patients with breast cancer.^27,28^ Improving this QI would require institutional funding to implement immunohistochemical testing at our center. In the interim, we plan to develop association with other public sector laboratories where such testing facilities may be available. Only HER2 testing was not performed in 13 patients, anticipating that they would not afford trastuzumab as a part of treatment. Our national breast cancer management guideline does not recommend HER2 testing as a part of standard evaluation protocol in breast cancers.^29^ This is consistent with the core level of resource-stratified guidelines for LMIC, which includes basic cancer medicines and basic hormonal therapies only.

Improved adherence to some QI can only be realized through wider engagement with external stakeholders. For example, most patients are unable to afford trastuzumab even with biosimilars being available.^30^ Improving access to trastuzumab therapy would therefore require implementation of a nation-wide strategy like price control or additional governmental funding support for these drugs. Lack of affordable stay facilities for the long duration of treatment is a cause of noncompliance. We plan to liaise with governmental and nongovernmental agencies to improve availability of affordable hostels or dormitories where patients along with their caregivers can stay during their treatment. Studies have shown poorer prognosis for patients with delayed diagnosis and initiation of treatment in patients with breast cancer.^31,32^

We have excluded some QIs related to pretreatment workup, surgery, and follow-up strategies by consensus. Magnetic resonance imaging scan was excluded for cost issues and lack of availability. A genetic counselor is not available in the hospital, and hence, required counseling is done by treating physicians themselves. For surgery QIs, single operation for noninvasive cancer was excluded because of a very low eligible percentage of patients in the group. Sentinel lymph node dissection is not performed at our center because of unavailability of related equipment. QIs for counseling, follow-up, and rehabilitation were also not included.

In addition, some other limitations are noteworthy. This is a retrospective study with a limited sample size, and QIs were audited for patients who took treatment at a single center. A national- or regional-level study is required to understand the quality of care that all patients with breast cancer receive. Follow-up is also short, and therefore, outcome data are immature. QI related to follow-up and rehabilitation will need separate auditing. However, these data have helped us to identify key institutional mitigation strategies, which need to be prospectively implemented to improve adherence to QI.

In conclusion, as per resource-stratified guidelines for breast cancer, we are now achieving the minimal target level for LMICs. With the phase-wise implementation of mitigation strategies discussed, we will try to improve the quality of breast cancer care in our hospital. A prospective quality improvement study is being planned for this. We have formulated a hospital-based guideline for breast cancer management in which these QIs have been incorporated.
